# Correction: Ferri et al. Low-Density Lipoprotein Cholesterol-Lowering Drugs: A Narrative Review. *J. Clin. Med.* 2024, *13*, 943

**DOI:** 10.3390/jcm13164582

**Published:** 2024-08-06

**Authors:** Nicola Ferri, Massimiliano Ruscica, Sergio Fazio, Alberto Corsini

**Affiliations:** 1 Department of Medicine (DIMED), University of Padova, 35122 Padova, Italy; 2 Veneto Institute of Molecular Medicine (VIMM), 35129 Padua, Italy; 3 Department of Pharmacological and Biomolecular Sciences “Rodolfo Paoletti”, University of Milan, 20133 Milan, Italy; alberto.corsini@unimi.it; 4 Department of Cardio-Thoracic-Vascular Diseases, Foundation IRCCS Cà Granda Ospedale Maggiore Policlinico, 20122 Milan, Italy; 5 Regeneron Pharmaceuticals Inc., Tarrytown, NY 10591, USA; sergio.fazio@regeneron.com

## Text Correction

There was an error in the original publication [1]. No dose adjustment is necessary in patients with mild hepatic impairment (Child–Pugh A), while its use is not recommended in patients with moderate (Child–Pugh B) or severe (Child–Pugh C) hepatic impairment [127].

A correction has been made to 6. Bempedoic Acid, 6.3. Pharmacokinetics:

No dose adjustment is necessary in patients with mild or moderate hepatic impairment (Child–Pugh A or Child–Pugh B). Since no data are available in patients with severe hepatic impairment (Child–Pugh C), they should undergo periodic liver function tests.

References

The original reference [127] has become [132]. Thus, all the references between [127] and [132] have been renumbered accordingly.

The new references are as follows:

127. De Filippo, O.; D’Ascenzo, F.; Iannaccone, M.; Bertaina, M.; Leone, A.; Borzillo, I.; Ravetti, E.; Solano, A.; Pagliassotto, I.; Nebiolo, M.; et al. Safety and efficacy of bempedoic acid: A systematic review and meta-analysis of randomised controlled trials. *Cardiovasc. Diabetol*. **2023**, *22*, 324. https://doi.org/10.1186/s12933-023-02022-z.

128. Ray, K.K.; Bays, H.E.; Catapano, A.L.; Lalwani, N.D.; Bloedon, L.T.; Sterling, L.R.; Robinson, P.L.; Ballantyne, C.M.; Trial, C.H. Safety and Efficacy of Bempedoic Acid to Reduce LDL Cholesterol. *N. Engl. J. Med.* **2019**, *380*, 1022–1032. https://doi.org/10.1056/NEJMoa1803917.

129. Singh, J.A.; Reddy, S.G.; Kundukulam, J. Risk factors for gout and prevention: A systematic review of the literature. *Curr. Opin. Rheumatol.* **2011**, *23*, 192–202. https://doi.org/10.1097/BOR.0b013e3283438e13.

130. Ray, K.K.; Nicholls, S.J.; Li, N.; Louie, M.J.; Brennan, D.; Lincoff, A.M.; Nissen, S.E. Efficacy and safety of bempedoic acid among patients with and without diabetes: Prespecified analysis of the CLEAR Outcomes randomised trial. *Lancet Diabetes Endocrinol*. **2023**, *12*, 19–28. https://doi.org/10.1016/S2213-8587(23)00316-9.

131. Ferri, N.; Corsini, A. Mechanism of bempedoic acid induced cholelithiasis: A role for statins to limit this adverse effect? *Pharmacol. Res.* **2023**, *196*, 106900. https://doi.org/10.1016/j.phrs.2023.106900.

132. Nilemdo: Summary of Product Characteristics. 2023. Available online: https://www.ema.europa.eu/en/documents/product-information/nilemdo-epar-product-information_en.pdf (accessed on 1 February 2024).

With this correction, the order of some references has been adjusted accordingly. The authors state that the scientific conclusions are unaffected. This correction was approved by the Academic Editor. The original publication has also been updated.

## References

[1] Ferri N., Ruscica M., Fazio S., Corsini A. (2024). Low-Density Lipoprotein Cholesterol-Lowering Drugs: A Narrative Review. J. Clin. Med..

